# Neprilysin is a Mediator of Alternative Renin-Angiotensin-System Activation in the Murine and Human Kidney

**DOI:** 10.1038/srep33678

**Published:** 2016-09-21

**Authors:** Oliver Domenig, Arndt Manzel, Nadja Grobe, Eva Königshausen, Christopher C. Kaltenecker, Johannes J. Kovarik, Johannes Stegbauer, Susan B. Gurley, Dunja van Oyen, Marlies Antlanger, Michael Bader, Daisy Motta-Santos, Robson A. Santos, Khalid M. Elased, Marcus D. Säemann, Ralf A. Linker, Marko Poglitsch

**Affiliations:** 1 Division of Nephrology and Dialysis, Department of Medicine III, Medical University of Vienna, Vienna, Austria; 2 Department of Neurology, University Hospital Erlangen, Erlangen, Germany; 3 Department of Pharmacology and Toxicology, Wright State University, OH, USA; 4 Department of Nephrology, Medical Faculty, Heinrich Heine University, Duesseldorf, Germany; 5 Division of Nephrology, Department of Medicine, Duke University and Durham VA Medical Centers, Durham, NC 27710, USA; 6 Attoquant Diagnostics GmbH, Vienna, Austria; 7 Max-Delbrück-Center for Molecular Medicine (MDC), Berlin-Buch, Germany; 8 Department of Physiology and Biophysics, National Institute of Science and Technology in Nanobiopharmaceutics, Federal University of Minas Gerais, Belo Horizonte, MG, Brazil; 9 Institute of Cardiology, University Cardiology Foundation, Porto Alegre, RS, Brazil

## Abstract

Cardiovascular and renal pathologies are frequently associated with an activated renin-angiotensin-system (RAS) and increased levels of its main effector and vasoconstrictor hormone angiotensin II (Ang II). Angiotensin-converting-enzyme-2 (ACE2) has been described as a crucial enzymatic player in shifting the RAS towards its so-called alternative vasodilative and reno-protective axis by enzymatically converting Ang II to angiotensin-(1-7) (Ang-(1-7)). Yet, the relative contribution of ACE2 to Ang-(1-7) formation *in vivo* has not been elucidated. Mass spectrometry based quantification of angiotensin metabolites in the kidney and plasma of ACE2 KO mice surprisingly revealed an increase in Ang-(1-7), suggesting additional pathways to be responsible for alternative RAS activation *in vivo*. Following assessment of angiotensin metabolism in kidney homogenates, we identified neprilysin (NEP) to be a major source of renal Ang-(1-7) in mice and humans. These findings were supported by MALDI imaging, showing NEP mediated Ang-(1-7) formation in whole kidney cryo-sections in mice. Finally, pharmacologic inhibition of NEP resulted in strongly decreased Ang-(1-7) levels in murine kidneys. This unexpected new role of NEP may have implications for the combination therapy with NEP-inhibitors and angiotensin-receptor-blockade, which has been shown being a promising therapeutic approach for heart failure therapy.

The prevalence of chronic kidney disease (CKD) is increasing world-wide^1^ and constitutes a major health and economic burden of modern societies^2^. Over-activation of the classical RAS-axis composed of the vasoconstrictor Ang II and its receptor (angiotensin type 1 receptor; AT1R) represents a key pathogenic factor in the etiology of CKD^3^,^4^. Thus, the RAS constitutes an attractive target complex for the pharmacological treatment of CKD^5^,^6^. Recently, a novel branch of the RAS, termed alternative RAS-axis, has been described. Its constituents, Ang-(1-7) and the cognate receptor MAS, functionally counterbalance biological effects of the classical RAS-axis^7^,^8^. Moreover, accumulating evidence supports a cardio- and reno-protective role of this alternative RAS-axis^7^,^9^,^10^. The protective hormone Ang-(1-7) is generated by various enzymes with ACE2 being its major producer^11^,^12^,^13^ by converting Ang II to Ang-(1-7), therefore potently shifting the balance between the classical and the alternative RAS. ACE2 deficiency results in hypertension and exaggerated renal and cardiovascular diseases^10^,^14^,^15^. Besides ACE2, other enzymes such as prolyl-carboxy-peptidase (PCP)^16^, prolyl-endo-peptidase (PEP)^17^ and Neprilysin (NEP, neutral endopeptidase)^18^ have been described, that are biochemically capable of producing Ang-(1-7) from Ang II and Ang I, respectively. Importantly, their actual contribution to renal Ang-(1-7) has not been elucidated, yet.

Since the combined pharmacologic blockade of NEP and AT1-receptor with LCZ696 showed significantly reduced overall mortality in the heart failure trial PARADIGM-HF^19^, we were especially interested in the involvement of NEP in renal alternative RAS activation. The underlying mechanism of LCZ696 was attributed to the stabilization of vasoactive peptides including bradykinin and natriuretic peptides via NEP blockade^20^. Importantly, the PARAMOUNT trial^21^ also observed an increase in urinary albumin secretion in LCZ696 treated patients raising questions about the involvement of NEP in kidney physiology and suggesting the presence of tissue specific physiologically relevant actions of LCZ696. We speculated that NEP inhibition might affect the local RAS in the kidney at the level of the alternative pathway, possibly providing a molecular explanation for clinical observations described above. Since CKD has been shown to be the most common co-morbidity in chronic heart failure^22^, proving this hypothesis is of major clinical importance and might affect future therapeutic strategies in the treatment of heart failure.

The investigation of the tissue localized RAS is technically challenging, which is reflected by the heterogeneity of reported tissue angiotensin concentrations. The fluctuation of Ang II in plasma ranges over 3 orders of magnitude^23^,^24^,^25^ while actual physiological levels range between 10 to 100 pg/ml^26^,^27^,^28^. To overcome some of the previous technical limitations, we used chemically identical isotope-labelled peptides as internal standards^29^,^30^ for the multiplex liquid chromatography-tandem mass spectrometry (LC-MS/MS) based quantification of 10 angiotensin metabolites in plasma and tissue^27^,^31^. This highly specific method allowed us to investigate RAS metabolic pathways and circulating as well as equilibrium angiotensin concentrations at a new level of detail. We characterized RAS enzymes and peptide metabolites in wild-type and ACE2 knockout (ACE2 KO) mice, being a well described model for studying the alternative RAS^14^,^15^. In order to assess potential clinical/therapeutic implications of our findings in murine tissue, we extended our angiotensin metabolic analyses to biopsies of living human kidney transplant donors.

## Results

### Increased RAS activity and elevated Ang-(1-7) levels in the ACE2 KO kidney

**Figure 1:**
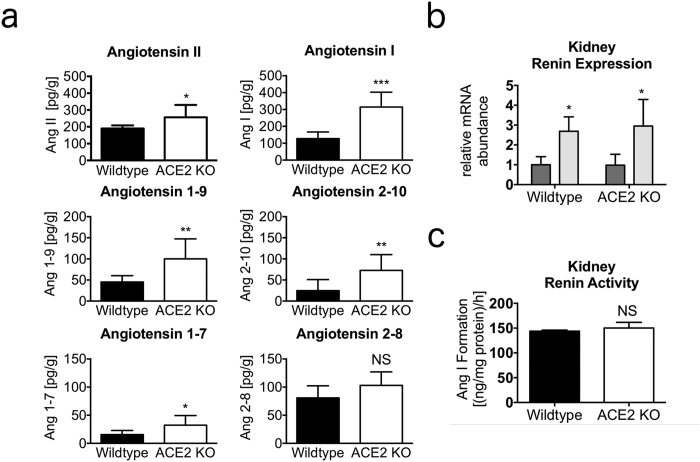
Elevated Ang-(1-7) levels in kidneys of ACE2 KO mice. (**a**) Kidney angiotensin concentrations of wild-type (C57BL/6) and ACE2 knockout (ACE2 KO) mice (pg/g gram net weight). *n* = 8 mice per group. Data presented as mean ± s.d. Two-tailed Student’s *t*-test. *P < 0.05 **P < 0.01 ***P < 0.001 vs. wild-type. **(b)** Relative mRNA abundance (to beta actin) of renin in murine renal medulla (dark grey) and cortex (light grey) of wild-type and ACE2 KO. *n* = 8 mice per group. Data presented as mean ± s.d. One-way analysis of variance (ANOVA). *P < 0.05 vs. medulla. **(c)** Renal renin activity of wild-type (black) ACE2 KO (white) assayed by Ang I formation determination in homogenates following recombinant murine angiotensinogen spiking. *n* = 4 mice per group. Data presented as mean ± s.d. Two-tailed Student’s *t*-test. not significant (NS) vs. wild-type.

Kidney angiotensin concentrations of ACE2 knockout (ACE2 KO) and wild-type littermate control mice were quantified by LC-MS/MS. A highly significant increase (p < 0.001) of angiotensin I (Ang I) was observed in ACE2 KO mice, which was accompanied by elevated concentrations of its downstream metabolites including Ang II (p = 0.027) and Ang-(1-7) (p = 0.023) (Fig. 1a). The ratios Ang-(1-7)/Ang I and Ang-(1-7)/Ang II were not significantly altered (Table 1).

The quantification of circulating (Supplementary Fig. 1a) and equilibrium (Supplementary Fig. 1b) angiotensin levels in murine plasma revealed increases for Ang I, Ang II, Ang-(2-8), Ang-(3-8) and Ang-(1-5) in ACE2 KO animals, pointing to the enhancement of the RAS at the level of plasma renin activity.

The abundance of angiotensins in immediately stabilized plasma was lower than in kidney, which is in line with previously published reports^32^. Equilibrium angiotensin levels in plasma were generally higher showing a strong correlation with circulating angiotensin levels. Comparison of equilibrium and circulating angiotensin levels revealed a significant increase of the Ang II/Ang I ratio.

As angiotensin quantification suggested an increase in plasma renin activity (PRA), we determined the concentration of AGT and plasma-renin-activity (PRA) by LC-MS/MS based Ang I formation assays. Plasma AGT was not altered in ACE2 KO mice (Supplementary Fig. 1c), while PRA was strongly elevated as compared to wild-type controls (31.9 [(ng/ml)/h] versus 14.5 [(ng/ml)/h], *n* = pool of 3 plasma samples). We further analysed the number of renin transcripts in the kidney by RT-PCR and determined the activity of renin in kidney homogenates, showing that neither renin expression (Fig. 1b) nor renin activity (Fig. 1c), were altered in ACE2 KO mice. We concluded that the increased plasma renin activity was likely due to posttranscriptional or posttranslational mechanisms such as altered secretion, since renal renin mRNA abundance as well as renin activity in kidney were unchanged in ACE2 KO mice.

### In murine kidneys, Ang II is primarily converted to Ang-(1-7) via PCP, but not ACE2

**Figure 2:**
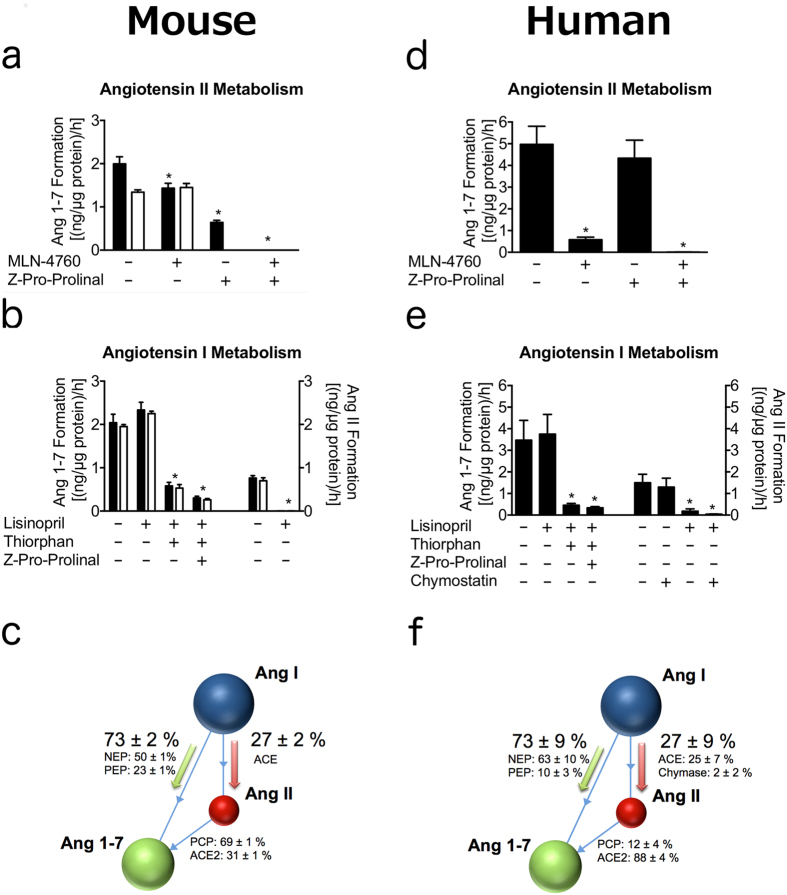
Analysis of angiotensin metabolism in kidney homogenates of mice **(a–c)** and human **(d–f ).** (**a**) Renal Ang II turnover to Ang-(1-7) of wild-type (black) and ACE2 KO mice (white) in presence and absence of specific inhibitors. *n* = 4 per group. Data presented as mean ± s.d. One-way analysis of variance (ANOVA). *P < 0.001 within wild-type group vs. solvent control. **(b)** Ang I turnover to Ang-(1-7) (left) and Ang II (right) of wild-type (black) and ACE2 KO (white). *n* = 4 per group. Data presented as mean ± s.d. One-way analysis of variance (ANOVA). *P < 0.001 vs. solvent control. **(c)** Enzymatic contribution to Ang II or Ang-(1-7) formation in mice was calculated on the inhibitor sensitive angiotensin formation rate of fig. 2a,b. Data presented as mean ± s.d. **(d)** Human renal Ang II turnover to Ang-(1-7) in presence and absence of specific inhibitors. *n* = 5 per group. Data presented as mean ± s.d. One-way analysis of variance (ANOVA). *P < 0.001 vs. solvent control. **(e)** Human Ang I turnover to Ang-(1-7) (left) and Ang II (right) in kidney homogenates. *n* = 5 per group. Data presented as mean ± s.d. One-way analysis of variance (ANOVA). *P < 0.001 vs. solvent control. **(f)** Enzymatic contribution to Ang II or Ang-(1-7) formation in human was calculated on the inhibitor sensitive angiotensin formation rate of fig. 2d,e. Data presented as mean ± s.d.

We used pharmacological inhibition in order to dissect the relative contribution of different Ang II metabolizing kidney proteases to Ang-(1-7) formation. Therefore, we spiked murine kidney homogenates with Ang II and analysed its conversion to Ang-(1-7) in the presence and absence of the ACE2 inhibitor MLN-4760^33^ and N-benzyloxycarbonyl-prolyl-prolinal (ZPP), the dual PCP and PEP inhibitor^17^,^18^ (Fig. 2a). As expected, the renal Ang-(1-7) formation from Ang II in wild-type was significantly higher compared to ACE2 KO (p = 0.007). MLN-4760 had no effect on Ang-(1-7) formation in the ACE2 KO kidney. However, it reduced Ang-(1-7) formation in wild-type mice to the level of ACE2 KOs. The additional presence of ZPP abolished Ang-(1-7) formation completely, assigning Ang-(1-7) formation primarily to PCP.

To better approximate the renal compartmentalization *in vivo*, we investigated if enzymatic distribution differs between cortex and medulla of murine kidneys (Supplementary Fig. 2). ACE2 mediated generation of Ang-(1-7) was higher in the cortex, whereas the relative contribution of ACE2 was still much lower compared to PCP, confirming a similar trend as observed in whole kidney homogenates (Fig. 2a).

The mRNA expression of all metabolically investigated enzymes was additionally determined in cortex and medulla of the kidney (Supplementary Fig. 3a). The expression of the Ang II degrading enzymes ACE2 and PCP were significantly higher in the cortex in both wild-type and ACE2 KO, supporting our previous observations in Ang II metabolic analysis (Supplementary Fig. 2). The relative abundances of Ang I cleaving enzyme mRNA (NEP, PEP, ACE) were quantified as being evenly spread.

### Ang II independent renal Ang-(1-7) formation via NEP in mice

Due to the equal expression and activity of the alternative Ang II to Ang-(1-7) forming enzyme PCP in wild-type and ACE2 KO, we decided to investigate Ang-(1-7) formation via alternative pathways to explain the increased kidney Ang-(1-7) levels in ACE2 KO. Other groups previously reported a direct conversion of Ang I to Ang-(1-7) by analysing isolated rat glomeruli *ex vivo*^34^.

Therefore, Ang-(1-7) and Ang II formation from Ang I was measured in murine kidney homogenates (Fig. 2b) in presence and absence of the ACE inhibitor lisinopril, ZPP and the NEP inhibitor DL-thiorphan. Due to the weak ACE inhibitory activity of DL-thiorphan^35^, we used DL-thiorphan only in combination with lisinopril for controlled complete ACE inhibition. As expected, wild-type and ACE2 KO mice showed high similarities in the relative abundance of these two pathways (Fig. 2b). Ang II formation from Ang I was efficiently blocked by lisinopril, while Ang-(1-7) formation remained unaffected (Fig. 2b). We concluded that Ang-(1-7) formation might rather be produced directly from Ang I than through Ang II as an intermediate, as previously suggested^36^. Importantly, 73.4 ± 2.1% of Ang I was converted directly to Ang-(1-7) while only 26.6 ± 2.1% were metabolized to Ang II, which could be further processed by ACE2 or PCP. We concluded that the elevated renal Ang-(1-7) levels in ACE2 KO resulted from the increased Ang I.

DL-thiorphan reduced Ang-(1-7) formation from Ang I by 75.2 ± 1.8% suggesting NEP to be the major source of Ang-(1-7) formation in the murine kidney. The dual prolyl-carboxy-peptidase/prolyl-endo-peptidase inhibitor ZPP resulted in a further significantly reduced Ang-(1-7) formation, however, to a much lower extent. Of note, triple treatment inhibiting ACE (lisinopril), NEP (DL-thiorphan) and PEP (ZPP) did not abolish Ang-(1-7) from Ang I completely, suggesting the presence of an alternative pathway possibly involving the metabolite Ang-(1-9).

### Prominent role of NEP in Ang-(1-7) formation in the human kidney

In order to assess potential clinical implications, we extended the metabolic analyses to kidney biopsies of living human transplant donors, observing several differences between mice and humans.

First, in contrast to mice, ACE2 dominantly contributed to formation of Ang-(1-7) from Ang II in human kidneys (88.2 ± 4.3% vs. 30.9 ± 0.3%) (Fig. 2d). Secondly, the ACE inhibitor lisinopril was not able to completely abolish Ang II formation in the human kidney. At least a part of ACE independent Ang II formation was blocked by the chymase inhibitor chymostatin, supporting a role for chymase in Ang II formation in the human kidney (Fig. 2e). However, the turnover from Ang I to Ang-(1-7) was reduced significantly in the presence of DL-thiorphan in both humans and mice, confirming the species-independent prominent role of NEP in renal Ang-(1-7) formation.

Human kidney biopsies subjected to angiotensin metabolic analysis were further analysed regarding the tissue localization of angiotensin metabolizing enzymes by immunohistochemistry (Supplementary Fig. 4). Glomerular epithelial cells displayed strong signals for NEP, while ACE2 expression appeared to be less abundant in these cells. This expression pattern shows that NEP and its blood-derived substrate Ang I are in local proximity *in vivo*, suggesting a role for NEP in Ang-(1-7) formation in the intact human kidney.

### Ang I dependent Ang-(1-7) formation primarily in renal cortex of mice

**Figure 3:**
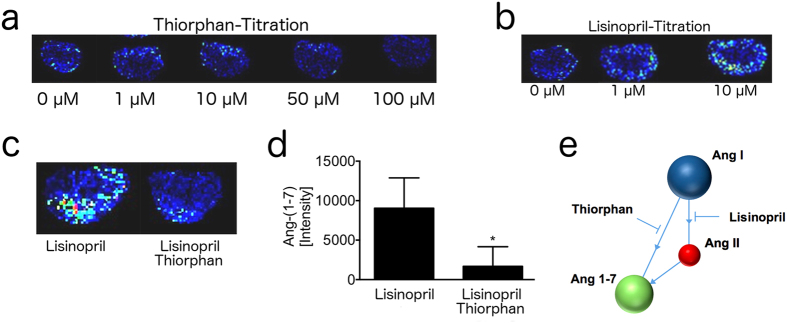
MALDI-Imaging reveals DL-thiorphan-sensitive Ang-(1-7) formation in renal cortex of mice. **(a)** Diminishing effect of increasing DL-thiophan concentrations (0 μM – 100 μM) on local turnover of Ang I to Ang-(1-7) is shown by MALDI-Imaging. Brighter colors of the murine kidney section indicate pronounced Ang-(1-7) formation in the renal cortex. **(b)** Increasing Ang-(1-7) signals depending on lisinopril concentration (0 μM–10 μM). **(c)** Inhibitory capacity of 100 μM DL-thiorphan on Ang I to Ang-(1-7) turnover in presence of 10 μM lisinopril **(d)** Intensity of Ang-(1-7) formation (Fig. 3c) was calculated and is given in bars. *n* = 4 per group. Data presented as mean ± s.d. Two-tailed Student’s *t*-test. *P < 0.05 vs. 10 μM lisinopril. **(e)** Schematic graph highlights the targeted angiotensin pathways of the used inhibitors.

To complement our analysis in homogenates with more structurally intact tissue, we analysed the Ang I metabolism to Ang-(1-7) in cryo-sections of whole murine kidneys by MALDI-Imaging^37^. We obtained a dose-depended DL-thiorphan-sensitive Ang-(1-7) signal in the cortex of murine kidney slices that were incubated with Ang I as a substrate (Fig. 3a). MALDI imaging tracks NEP activity by measuring the intensity of Ang-(1-7) signals on the surface of tissue slices that are incubated with an excess of Ang I as the natural substrate. In order to enhance the readout signal, protease inhibitors are added to the incubation buffer, that are aimed to avoid the degradation of Ang-(1-7) generated during the incubation. We observed a concentration dependent increase of Ang-(1-7) in the presence of lisinopril (Fig. 3b). As the stabilization of the product of the investigated enzymatic reaction is improving the signal in MALDI imaging, we decided to include lisinopril into MALDI assay. Beside blocking the ACE mediated degradation of Ang-(1-7)^38^, the ACE inhibitor lisinopril prevents formation of Ang-(1-7) via Ang II (Fig. 3e). The specificity of the obtained Ang-(1-7) signal for NEP was confirmed by the NEP inhibitor DL-thiorphan, which was added on top of lisinopril (Fig. 3c,d). We observed a significant reduction of Ang-(1-7) formation from Ang I (p = 0.045) suggesting a role of NEP in Ang-(1-7) formation in the intact murine kidney, with an increased enzymatic density in the renal cortex.

### NEP inhibition by LBQ657 (sacubitrilat) reduced renal Ang-(1-7) levels *in vivo*

Mice were treated intraperitoneally with 50 mg/kg of the NEP inhibitor LBQ657 in order to investigate the impact of short term NEP inhibition on kidney angiotensin levels *in vivo*. Administration of LBQ657 significantly reduced Ang-(1-7) levels in the murine kidney by 53.1 ± 10.6% (p = 0.026) accompanied by a lowered Ang-(1-7)/Ang I ratio (p = 0.047) (Table 2). No significant alteration of any other quantified angiotensin could be observed in the LBQ657 treated group, although a clear trend towards reduced levels of angiotensins was present. Mean values for plasma equilibrium Ang-(1-7) levels and the Ang-(1-7)/Ang I ratio were also reduced in LBQ657 treated animals (Table 3). However, p-values remained clearly above the significance threshold of 0.05.

## Discussion

In this study, we dissected enzymatic Ang-(1-7) formation in the murine and human kidney, combining genetic knockout and pharmacological inhibition with multiplex mass spectrometry (RAS fingerprint) and refined MALDI-Imaging. Characterizing the RAS in ACE2 knockout mice by RAS fingerprint revealed increased levels of Ang I, Ang II and their metabolites in comparison to wild-type mice. This is in line with previous reports, describing increased levels of Ang II in ACE2 KO mice^39^,^40^,^41^. We and others further detected increased renal concentrations of Ang-(1-7) in ACE2 KO mice^15^, which seems to undermine the role of ACE2 in Ang-(1-7) formation in the kidney. Our findings point to the conclusion that the elevated Ang II levels in ACE2 KO animals are more likely a result of the increased plasma renin than being a consequence of the reduced Ang II breakdown by the lack of ACE2. Multiple mechanisms are known to be involved in the control of renin secretion including salt and fluid homeostasis, sympathetic nervous system activity, blood pressure and hormonal status^42^. The mechanisms lying behind the up-regulation of renin in response to ACE2 KO still need to be investigated and might involve ACE2 targets beyond the angiotensin system potentially interfering with renin secretion. Comparing Ang-(1-7)/Ang II ratios between kidneys of wild-type and ACE2 KO mice (Table 1), challenges the classical concept that Ang-(1-7) is primarily generated via cleavage of Ang II by ACE2^13^,^43^,^44^. Undoubtedly, the knockdown of ACE2 has detrimental consequences on kidney^45^ and lung^46^ physiology and has been reported to be involved in the development of diabetes-induced cardiovascular complications^47^ and inflammation^48^. But only few and controversial data are available connecting ACE2 KO with actual angiotensin concentrations, which might be in part caused by technical issues in sample processing and subsequent angiotensin quantification. One could speculate that ACE2 generates Ang-(1-7) especially in proximity to its cognate receptor in a local molecular environment, which could explain the lack of impact on total plasma and tissue Ang-(1-7) levels. Beside Ang II conversion in Ang-(1-7), ACE2 is involved in apelin signaling^49^, bradykinin metabolism and dynorphin A^50^ degradation, as well as tryptophan uptake in the gut via a non-enzymatic mechanism^41^, which might affect physiological conditions beyond RAS signaling.

Based on our observations in ACE2 KO mice, we decide to investigate Ang-(1-7) formation via alternative pathways and found NEP as a major renal Ang-(1-7) forming enzyme via Ang I cleavage. We further compared the relative metabolic rates in kidney homogenates and revealed species-specific similarities and differences. The relative formation rate of Ang II and Ang-(1-7) after Ang I spike was similar between humans and mice. However, the contribution of NEP in Ang-(1-7) formation was slightly increased in humans. Chymase activity was detected in the human kidney only, which is in line with previous findings^51^. Interestingly, the relative contribution of ACE2 to Ang-(1-7) formation was more prominent in human compared to murine kidneys. Investigation of local turnover of Ang I on structurally intact kidneys using MALDI-Imaging showed a prominent local Ang-(1-7) generation in the renal cortex. Moreover, Ang II spiking experiments in separated cortex and medulla homogenates (Supplementary Fig. 2) revealed a higher Ang-(1-7) formation rate in the renal cortex indicating a crucial role of Ang-(1-7) in this compartment.

We are aware that the investigation of enzyme activities in tissue homogenates are not reflecting the *in vivo* situation as tissue homogenization releases multiple proteolytic enzymes from intracellular compartments facilitating non-physiological proximity of substrates and enzymes. In order to abrogate the impact of potentially interfering proteolytic enzymes on the assay readouts, specific inhibitor cocktails were designed to inhibit these interfering proteases, while preserving the activity of the enzymes of interest, which was proven by the usage of the recombinant enzymes and their specific inhibitors. While lisinopril and MLN-4760 are supposed being specific inhibitors for ACE and ACE2, respectively, chymostatin inhibits a broad range of enzymes, such as chymase, chymotrypsin and lysosomal cysteine proteinases (cathepsins, A, B, C, G, H, L). Importantly, the NEP inhibitor DL-thiorphan has been reported to possess ACE inhibitory activity^35^. In fact, DL-thiorphan mediated inhibition of ACE was one of the reasons why NEP activity in MALDI imaging and tissue homogenates was investigated in the presence of lisinopril, as complete blockade of ACE by lisinopril prevents DL-thiorphan mediated effects on Ang-(1-7) stability.

Aiming to proof the *in vivo* relevance of our observations obtained in homogenate analysis and MALDI imaging, we treated mice by intraperitoneal injection with the specific active NEP inhibitor LBQ657 (sacubitrilat) and measured endogenous renal angiotensin concentrations. Endogenous angiotensin levels in kidneys and plasma of i.p. treated animals (Table 2 and Table 3) were elevated compared to untreated mice (Fig. 1, Supplementary Fig. 1a/b). In order to achieve the required dose for *in vivo* treatment of mice with LBQ657, that has been previously chemically produced by alkaline hydrolysis (Supplementary Fig. 5) of AHU-377 (sacubitril), 50% DMSO had to be used as a vehicle, which was different to the experiment in which we compared ACE2 KO and wild-type mice in terms of angiotensin levels. We speculated that increased angiotensin levels observed in vehicle treated animals might be explained by DMSO mediated hemodynamic changes, since previous telemetry experiments revealed a temporary decrease in blood pressure in mice following DMSO administration (data not shown).

We further observed a general trend towards decreased renal angiotensin metabolite levels following NEP inhibitor administration suggesting differences in kidney renin activity. Therefore, we used the Ang-(1-7)/Ang I ratio as a surrogate measure for NEP activity in the kidney that is independent of overall RAS activity. However, a potential interference of NEP inhibition on overall kidney RAS activity cannot be excluded and needs to be investigated in further studies.

Importantly, we could confirm that the administration of the NEP inhibitor LBQ657 significantly reduced Ang-(1-7) levels in the murine kidney, accompanied by a significant decrease in the Ang-(1-7)/Ang I ratio. (Table 2). However, the reducing effect on Ang-(1-7) formation appeared to be less prominent *in vivo* when compared to previous findings in tissue homogenates. Beside NEP, further enzymes seem to be significantly involved in the generation of renal Ang-(1-7) *in vivo* as NEP inhibition by LBQ657 could not completely abolish Ang-(1-7) levels. Importantly, the lack of the reduction in the Ang-(1-7)/Ang II ratio in ACE2 KO mice suggests the existence of other alternative pathways of Ang-(1-7) formation that may compensate for the lack of ACE2. It might be speculated that ACE2 KO results in an enzymatic rearrangement in the kidney, where NEP or PCP could take over the role of ACE2 in Ang-(1-7) formation. If PCP might solely be able to compensate for ACE2 mediated Ang-(1-7) formation needs to be investigated in further studies on ACE2 knockout animals treated with NEP inhibitors.

LC-MS/MS based analysis of equilibrium angiotensin levels in plasma was employed to characterize the soluble RAS in murine samples. Equilibrium angiotensin levels make use of the fact that angiotensinogen is present in plasma at high concentrations, providing a stable rate of Ang I formation over a certain *ex vivo* incubation period, which is also the basis for the determination of plasma renin activity (PRA). In contrast to PRA assays, where Ang I is stabilized by using appropriate protease inhibitors, in equilibrium analysis Ang I is immediately converted to further downstream angiotensin metabolites and equilibrium levels are established. These levels are characterized by equal formation and degradation rates of individual angiotensin metabolites in the plasma sample that are determined by all enzymes, which are involved in plasma angiotensin metabolism. This principle is valid for all resulting angiotensin metabolite equilibrium levels and their ratios to be a valid surrogate of RAS enzyme activities in a plasma sample. Equilibrium angiotensin levels are generally higher compared to levels obtained from samples collected using an appropriate protease inhibitor cocktail for immediate inhibition of angiotensinases during sampling (Supplementary Fig. 1), which has also been observed in previous studies in humans and rats^27^,^38^. Interestingly, equilibrium angiotensin levels show a high correlation with immediately stabilized angiotensin levels. One could speculate that the increased equilibrium levels might be caused by the absence of endothelial receptors and enzymes during the *ex vivo* incubation step, therefore suggesting equilibrium angiotensin levels represent a portion of angiotensins that is visible to endothelial surfaces.

Although the trends on reduced Ang-(1-7) levels under NEP inhibition in plasma equilibrium analysis were similar compared to angiotensin levels in kidneys, the effects did not reach significance. A possible explanation for the more prominent effect in the kidney could be the difference in NEP abundance, since NEP is a membrane- and blood cell bound enzyme, highly expressed in the kidney while hardly detectable in plasma^52^. As NEP has been reported being expressed on leucocytes further investigations using whole blood are required to highlight NEP influence on systemic renin-angiotensin-system.

However, in the context of RAS-interfering therapies, our findings indicate major biochemical and physiological differences between human and murine kidneys. The formation of the key effector molecules of the classical and the alternative RAS (Ang II and Ang-(1-7), respectively), was significantly different between humans and mice. The role of ACE2 in human kidney function might be underestimated by the investigation of murine model systems, where ACE2 seems to be less involved in Ang II metabolism compared to humans. We conclude that species-specific molecular differences within the RAS exist between mice and humans that could affect kidney physiology and should be considered for future translational therapeutic approaches.

Our findings could be of clinical relevance for patients treated with an ACE inhibitor. ACE inhibitor treatment results in an increase of Ang I by inhibiting its conversion to Ang II^27^,^53^ and shows significant benefits in CKD^1^,^4^,^5^. The increased availability of Ang I as a substrate for NEP in the kidney may have a significant impact on Ang-(1-7) formation *in vivo*. While Ang I levels are elevated and Ang II as a substrate for ACE2 or PCP mediated formation of Ang-(1-7) is less available^27^, NEP is likely to take over a major role in Ang-(1-7) formation. In this context, the combined inhibition of ACE and NEP, despite showing remarkable effects on blood pressure^54^, might be detrimental in therapeutic settings, where Ang-(1-7) mediates renoprotective effects^9^. The presence of chymase in human kidneys suggests a bypass of the inhibitory effect of ACE inhibitors on Ang II levels in humans, which might gain importance in pathologic conditions like CKD, where cellular re-organization of the kidney structure occurs. Of note, it cannot be excluded that direct formation of Ang II from angiotensinogen via cathepsin G or tonin might also play a role in the maintenance of kidney Ang II levels *in vivo*. Nevertheless, ACE2 may antagonize this additional pathway of Ang II formation and may serve as efficient protection from excess Ang II in the human setting.

Our findings describe a role of NEP in the formation of the alternative, reno-protective Ang-(1-7)/MAS receptor axis in the kidney. This molecular mechanism may potentially affect the therapeutic administration of NEP inhibitor in combination with Ang II receptor blocker, LCZ696, which was recently approved for treatment of heart failure. The recent heart failure trial PARADIGM-HF involving over 8,000 patients had to be halted, since LCZ696 dramatically reduced overall mortality compared to treatment with valsartan^19^. Beyond Ang II blockade, the underlying mechanisms of these profound effects of LCZ696 are attributed to the stabilization of bradykinin and natriuretic peptides by NEP inhibition. However, data from the PARAMOUNT trial further revealed an increase in urinary albumin to creatinine ratio (UACR) in the LCZ696 treated group in patients with moderately impaired kidney function^21^ indicting an involvement of NEP in kidney physiology, which could either be contributed by the direct effects of natriuretic peptides or to changes in local angiotensin metabolism.

Taking our findings together with results from clinical trials, the therapeutic inhibition of NEP in heart failure patients, especially those suffering from renal co-morbidities, may require further investigation.

## Materials and Methods

### Mice Study

All animal procedures were approved by the federal state government of middle Frankonia (#TS-05/10, #54-2532.1-12/11, AZ 84-02.04.2012.A250) in accordance to the German animal protection law and were performed by skilled experimenters.

### Murine Kidney and Plasma Preparation (wild-type vs. ACE2 KO)

12 weeks old female wild-type and ACE2 KO mice with C57BL/6j background were maintained at the animal care facility of the University Erlangen-Nuremberg, Germany under a 12 h light/dark cycle, normal chow (SNIFF, Soest) and tap water *ad libitum*. For dissection of kidneys, mice were deeply anaesthetized, then CO2 asphyxiated and transcardially perfused with PBS. Harvested kidneys were immediately snap-frozen in liquid nitrogen and stored at −80 °C until shipment for analysis. Obtained heparin plasma samples were either equilibrated for 30 min at 37 °C before stabilization for equilibrium analysis or directly stabilized using a preformulated peptidase inhibitor cocktail (Attoquant Diagnostics GmbH) for circulating angiotensin analysis^38^. Stabilized samples were snap-frozen and stored at −80 °C until further processing.

### AHU-377 (sacubitril) – LBQ657 (sacubitrilat)

The NEP inhibitor AHU-377 (sacubitril) is a prodrug and physiologically activated in the liver by CES1^55^. For intraperitoneal administration of the active drug LBQ657 (sacubitrilat), we chemically activated the prodrug by standard ester hydrolysis. 7-fold molar excess of NaOH was added to AHU-377 (Sigma-Aldrich) and incubated for 6 h at 60 °C. Solution was neutralized by equimolar hydrochlorid acid and evaporated to dryness. Chemical yield was controlled by mass spectrometry, operated in ES-negative mode (Supplementary Fig. 5).

### Murine Kidney and Plasma Preparation (Inhibitor Treatment)

10 to 12 weeks old female wild-type with C57BL/6j background were maintained at the animal care facility of the University Duesseldorf, Germany under a 12 h light/dark cycle, normal chew (SNIFF, Soest) and tap water *ad libitum*. Vehicle (50% DMSO) or NEP-inhibitor (LBQ657 (Sigma-Aldrich): 50 mg/kg) were administrated i.p. 4 hours prior to kidney and heparin plasma sampling. Kidneys were obtained as given above, while heparin plasma was incubated for 30 min at 37 °C for equilibrium analysis, stabilized^38^ and stored at −80 °C until shipment.

### Human Kidney Biopsy Preparation

This study was approved by the Ethics Committee of the Medical University of Vienna (EK #1496/2014) and was performed in accordance to the good clinical practice guidelines. Written informed consent was obtained before biopsies were taken perioperative from 5 kidney donors in 2014. Renal biopsies were either snap frozen in liquid nitrogen for angiotensin metabolism analysis or fixed in formalin for histological examination.

### Immunoreactive staining

Formalin-fixed paraffin-embedded myocardial biopsies were cut to 4 μm sections, deparaffinized in xylene and rehydrated in graded ethanol and water. Antigens were unmasked by autoclaving (30 min) in retrieval buffer (Chymase, NEP: Tris/EDTA buffer pH8; ACE, ACE2, PEP, PCP: Na-citrate buffer pH6) and endogenous peroxidase was blocked (3% H_2_O_2_). Following blockade of non-specific background (Ultra V Block, Thermo Scientific, MA), slides were incubated in primary antibody (ACE 1:50, PEP 1:500, PCP 1:300, all Sigma-Aldrich; chymase 1:500, abcam; NEP, 1:50, Novocastra; ACE2, 1:100, R&D Systems) diluted in 1% bovine serum albumin (BSA) in PBS for 1 hour. Subsequently, slides were washed in PBS, treated with primary antibody enhancer, HRP polymer and developed using 3,3′-diaminobenzidine enhanced by cobalt/nickelchloride (all Thermo Scientific) to form a brown/black precipitate. After counterstaining with haematoxylin (Merck, Germany), slides were dehydrated, coverslipped and photomicrographed.

### Analysis of Angiotensin Metabolism

Metabolic RAS analysis was performed by Attoquant Diagnostics GmbH, Vienna, Austria. Renal biopsies of mice and humans were extracted and homogenized in phosphate-buffered saline (PBS) using low-energy sonication. The metabolism of spiked Ang I, Ang II (Fisher Scientific) or recombinant murine AGT (Sino Biological Inc.) for the renin activity assay, respectively, was assayed in homogenates after *ex vivo* incubation at 37 °C in presence or absence of specific enzyme inhibitors. Lisinopril (10 μM, Sigma-Aldrich), chymostatin (10 μM, Sigma-Aldrich), Z-Pro-Prolinal (20 μM, Sigma-Aldrich), MLN-4760 (10 μM, Merck-Millipore), DL-thiorphan (100 μM, Sigma-Aldrich). Aminopeptidase Inhibitor (10 μM, Sigma-Aldrich) was added to all samples.

The enzyme activity was calculated by determining the inhibitor sensitive part of product formation in incubated samples and is presented as a product formation in ng of the corresponding angiotensin product per μg protein per hour. In order to proof linearity of the assay in dependence of enzyme abundance, Ang I and Ang II metabolism were assayed in serial diluted kidney homogenates (Supplementary Fig. 6). All experiments were performed in the linear range of the assay.

### Angiotensin Peptide Quantification

Angiotensin peptide quantification by liquid chromatography tandem-mass spectrometry analysis (LC-MS/MS) was performed by Attoquant Diagnostics GmbH (Vienna, Austria) as previously described^27^,^31^.

### MALDI-Imaging

Consecutive tissue sections were prepared from fresh frozen kidneys (*n* = 4) as previously described^37^ and incubated with 772 μM Ang I at 37 °C for 5 min. Inhibition of renal Ang-(1–7) formation was tested using reaction mixtures spiked with lisinopril (10 μM, Sigma-Aldrich) alone or in combination with the neprilysin-inhibitor DL-thiorphan (100 μM, Sigma-Aldrich) or Aminopeptidase-A inhibitor (10 μM, Sigma-Aldrich) was present in all samples. The imaging technique is described in detail elsewhere^37^.

### Real Time PCR

Relative abundance of ACE, ACE2, PEP, PCP, renin and NEP mRNAs in murine renal medulla and cortex were analysed by semiquantitative real time PCR. Kidneys were separated into medulla and cortex and further mechanically disrupted in TriFast^®^(PeqLab, Erlangen, Germany) using a glass douncer. Total RNA was extracted using the PeqGold RNA isolation kit (PeqLab) and 1 μg of RNA reversely transcribed using QuantiTect^®^ transcriptases (QIAGEN) and random polyT primers. Resulting cDNA was diluted (1:30) in DNase/RNase free water (Gibco) and directly used in amplification reactions. Predeveloped TaqMan probe/primer pair assays (Supplementary Fig. 4b) and accessory reagents (PCR SuperMix-UDG, ROX reference dye, all from Life Technologies) were used for amplification. PCR reactions were performed at a 10 μl scale on an Applied Biosystems SDS 7900 real time PCR System (Life Technologies) in quadruplicates; relative quantification was performed by the ΔΔCT method (Schmittgen and Livak 2008), normalizing target gene expression on *Actb*/β-Actin.

### Statistic

Unpaired Student’s *t-t*est was used to evaluate the differences between two groups. For more than two groups, ordinary one-way ANOVA followed by Bonferroni’s multiple comparison correction was used.

For real-time PCR, relative quantification values were tested for normal distribution using the D’Agostino & Pearson omnibus normality test and afterwards analysed by one-way ANOVA with Tukey’s test.

## Additional Information

**How to cite this article**: Domenig, O. *et al*. Neprilysin is a Mediator of Alternative Renin-Angiotensin-System Activation in the Murine and Human Kidney. *Sci. Rep.* **6**, 33678; doi: 10.1038/srep33678 (2016).

## Supplementary Material

Supplementary Information

## Figures and Tables

**Table 1:** Ratios of renal angiotensin metabolites (pg/g) in wild-type and ACE2 knockout (ACE2 KO) mice.

Angiotensin Ratio	Wild-type	ACE2 KO
Ang-(1-7)/Ang I (x100)	14.3 ± 10.5	11.0 ± 5.7
Ang-(1-7)/Ang II (x100)	8.4 ± 4.3	12.6 ± 4.9

Data are mean ± s.e.m. of *n* = 8.

**Table 2:** Renal angiotensin metabolites (pg/g) and their ratios of mice treated with vehicle or LBQ657 (50 mg/kg).

Angiotensin (pg/g)	Vehicle	LBQ657
Ang-(1-7)	74 ± 14	35 ± 4*
Ang II	952 ± 172	642 ± 99
Ang I	399 ± 69	277 ± 31
Ang-(1-9)	249 ± 62	162 ± 10
Ang-(2-8)	211 ± 33	195 ± 28
Ang-(2-10)	147 ± 22	89 ± 16
Ang-(1-7)/Ang I (x100)	18.4 ± 0.59	13.4 ± 2.0*
Ang-(1-7)/Ang II (x100)	8.1 ± 1.1	6.1 ± 1.2

Data are mean ± s.e.m. of *n* = 5.

*P < 0.05 vs. vehicle.

**Table 3:** Equilibrium plasma angiotensin metabolites (pg/ml) and their ratios of mice treated with vehicle or LBQ657 (50 mg/kg).

Angiotensin (pg/ml)	Vehicle	LBQ657
Ang-(1-7)	39 ± 12	20 ± 7
Ang II	1396 ± 294	1423 ± 540
Ang I	1061 ± 142	970 ± 341
Ang-(1-5)	84 ± 11	73 ± 34
Ang-(2-8)	252 ± 56	269 ± 91
Ang-(3-8)	101 ± 20	114 ± 36
Ang-(1-7)/Ang I (x100)	3.5 ± 0.8	1.8 ± 0.2
Ang-(1-7)/Ang II (x100)	3.7 ± 1.5	1.5 ± 0.2

Data are mean ± s.e.m. of *n* = 5.
