# Supplementary material for: Neprilysin is a Mediator of Alternative Renin-Angiotensin-System Activation in the Murine and Human Kidney
Source: Sci Rep. 2016 Sep 21;6:33678. doi: 10.1038/srep33678 (PMC5030486; doi:10.1038/srep33678)
Supplement: Supplementary Information [file srep33678-s1.pdf]

Supplementary Information

NEPRILYSIN IS A MEDIATOR OF ALTERNATIVE RENIN-ANGIOTENSIN-SYSTEM ACTIVATION IN THE MURINE AND HUMAN KIDNEY

Oliver Domenig[1#], Arndt Manzel[2#], Nadja Grobe[3], Eva Koenigshausen[4], Christopher C. Kaltenecker[1], Johannes J. Kovarik[1], Johannes Stegbauer[4], Susan B. Gurley[5], Dunja van Oyen[6], Marlies Antlanger[1], Michael Bader[7], Daisy Motta-Santos[8], Robson A.S. Santos[9], Khalid M. Elased[3], Marcus Säemann[1], Ralf A. Linker[2], Marko Poglitsch[6*]

[1]Division of Nephrology and Dialysis, Department of Medicine III, Medical University of Vienna, Vienna, Austria

[2]Department of Neurology, University Hospital Erlangen, Erlangen, Germany

[3]Department of Pharmacology and Toxicology, Wright State University, OH, USA

[4]Department of Nephrology, Medical Faculty, Heinrich Heine University, Duesseldorf, Germany [5]Division of Nephrology, Department of Medicine, Duke University and Durham VA Medical Centers, Durham, NC 27710, USA

[6]Attoquant Diagnostics GmbH, Vienna, Austria

[7]Max-Delbrück-Center for Molecular Medicine (MDC), Berlin-Buch, Germany

[8]Department of Physiology and Biophysics, National Institute of Science and Technology in Nanobiopharmaceutics, Federal University of Minas Gerais, Belo Horizonte, MG, Brazil

[9]Institute of Cardiology, University Cardiology Foundation, Porto Alegre, RS, Brazil

#Equally contributing authors

Correspondence to:

Marko Poglitsch, PhD

Attoquant Diagnostics GmbH

Vienna Biocenter 5

1130 Vienna

Austria

telephone: +43-1-8656577-121

fax: +43-1-8656577-800

e-mail: marko.poglitsch@attoquant.com

Supplementary Figure 1: Elevated plasma angiotensin levels in ACE2 KO mice

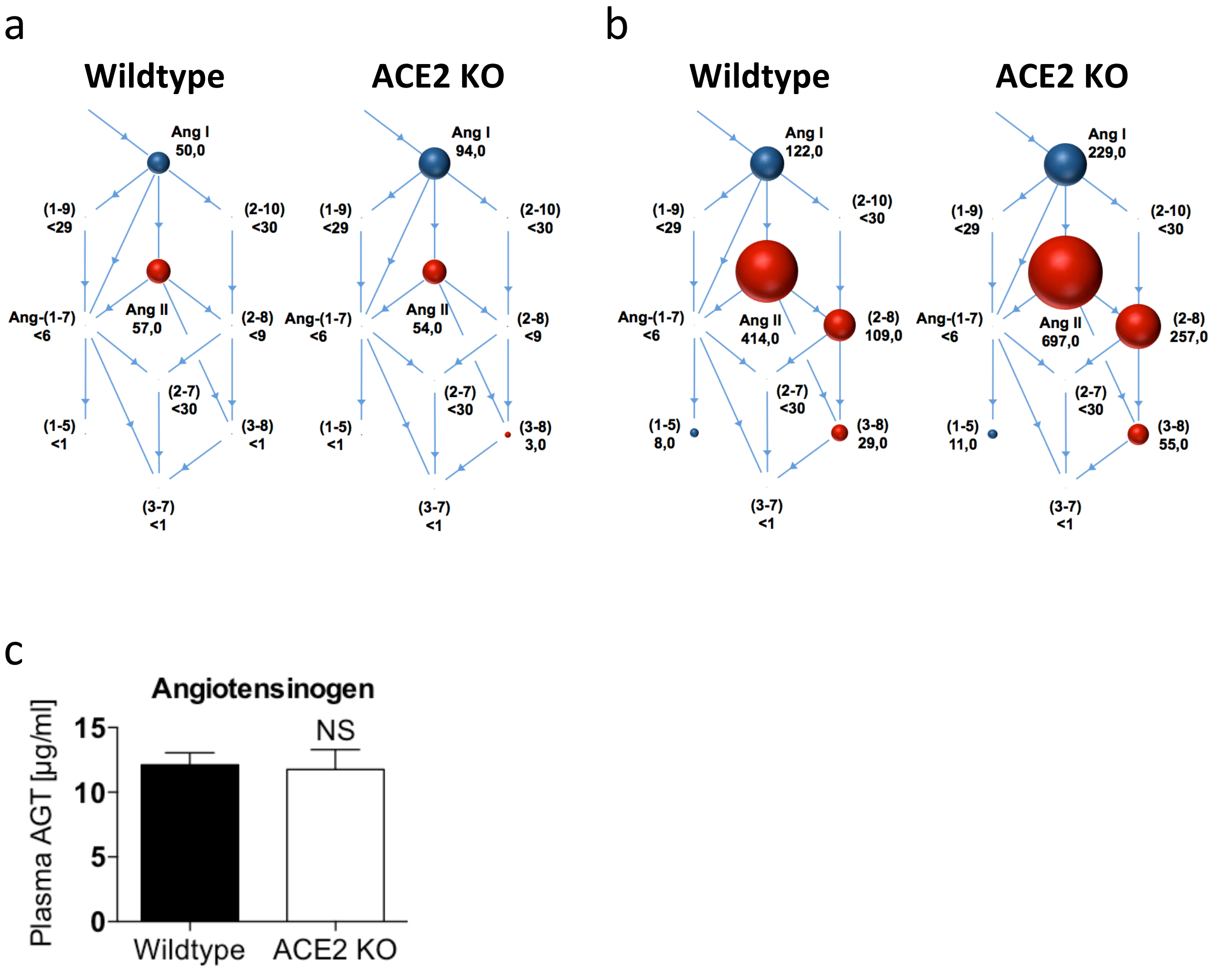

(a) Mean of immediatly stablized plasma angiotensin concentrations of wildtype (C57BL/6) and ACE2 knockout mice (ACE2 KO) are depicted as RAS-Fingerprints. The diameter of the spheres reflects the concentration of the respective peptide metabolite, which is also given in pg/gram net weight next to each individual sphere. The amino acid sequence of each angiotensin metabolite is schematically given in brackets beside the corresponding sphere. The sequence annotation is based on the decapeptide Ang I (1–10) which is N- or C-terminally cleaved. < indicates concentrations below the given quantification limits. Assumed metabolism pathways of peptides are illustrated by arrows connecting their substrate and product. *n*=4 mice per group. **(b)** Mean of equilibrated plasma angiotensin levels of wildtype and ACE2 KO mice are depicted as RAS-Fingerprints. **(c)** Plasma angiotensinogen levels of wildtype (black) and ACE2 KO (white). *n*=4 mice per group. Data presented as mean±s.d. Two-tailed Student's *t*-test. not significant (NS) vs. wildtype.

Supplementary Figure 2: ACE2 contributes to Ang-(1-7) formation primarily in the renal cortex

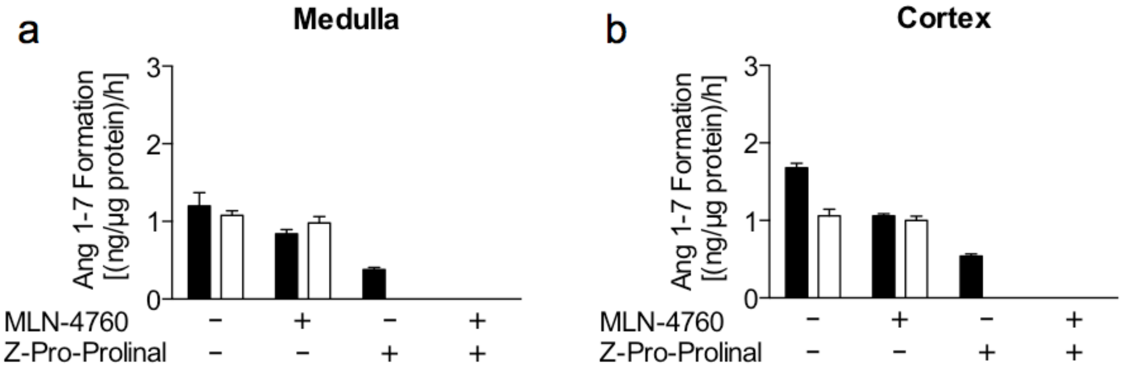

(a) Angiotensin II turnover to Ang-(1-7) in murine renal medulla homogenates of wildtype (black) and ACE2 KO (white) in presence and absence of specific inhibitors. *n*=2 pools of 3 murine homogenates. Data presented as mean±s.d. **(b)** Renal Ang II turnover to Ang-(1-7) in murine renal cortex homogenates of wildtype (black) and ACE2 KO (white) in presence and absence of specific inhibitors. *n*=2 pools of 3 murine homogenates. Data presented as mean±s.d.

Supplementary Figure 3: Expression profiling of Renin-Angiotensin-System related enzymes

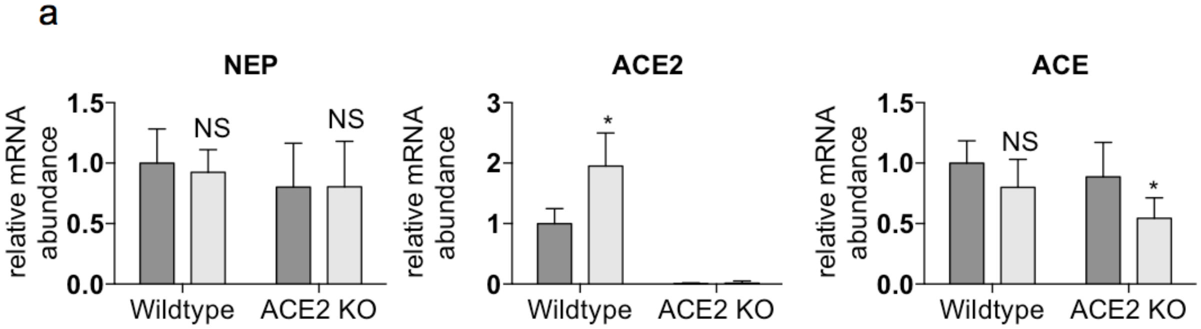

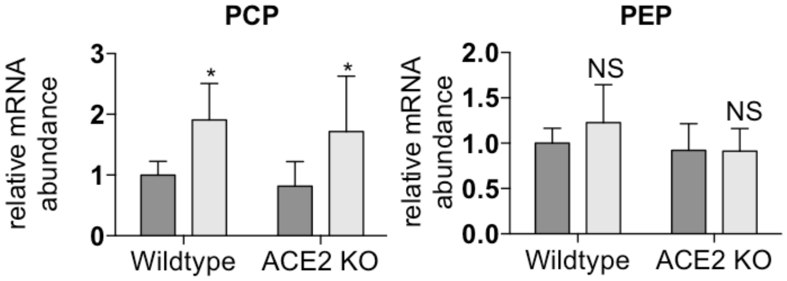

b

Target gene	Assay ID (Life technologies)
Actb/beta Actin	Mm00802048_m1
Ace/ACE	Mm00802048_m1
Ace2/ACE2	Mm01159003_m1
Mme/NEP	Mm00485028_m1
Prep/PEP	Mm00448377_m1
Prcp/PCP	Mm00804502_m1

(a) Relative mRNA (to beta actin) abundances of NEP, ACE2, ACE, PCP and PEP in renal medulla (dark grey) and cortex (light grey) of wildtype and ACE2 knockouts (ACE2 KO). *n*=8 mice per group. Data presented as mean±s.d. One-way analysis of variance (ANOVA). **P*<0.05 or NS (not significant) vs. medulla. **(b)** Assay ID (Life Technologies) of primer for the given enzymes.

Supplementary Figure 4: Immunoreactive staining for RAS enzymes in human kidney

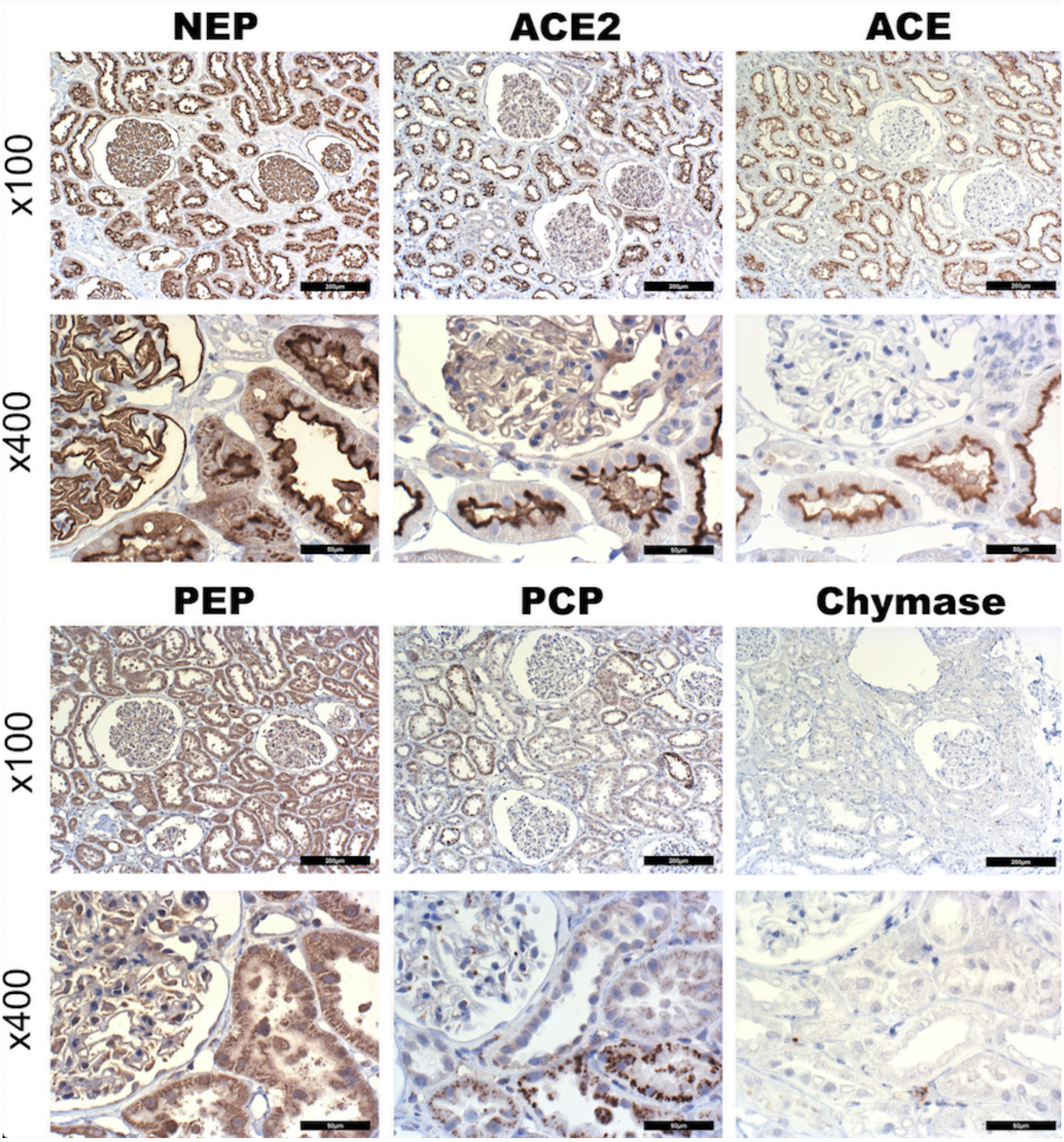

Immunoreactive staining for neprilysin (NEP), angiotensin converting enzyme 2 (ACE2), angiotensin converting enzyme (ACE), prolyl endo peptidase (PEP), prolyl carboxy peptidase (PCP) and chymase in living donor kidney biopsies in 100x and 400x magnitude. Scale bars are 200 µm and 50 µm, respectively.

Supplementary Figure 5: MS spectrum of AHU-377 and LBQ657

AHU-377 **LBQ657**

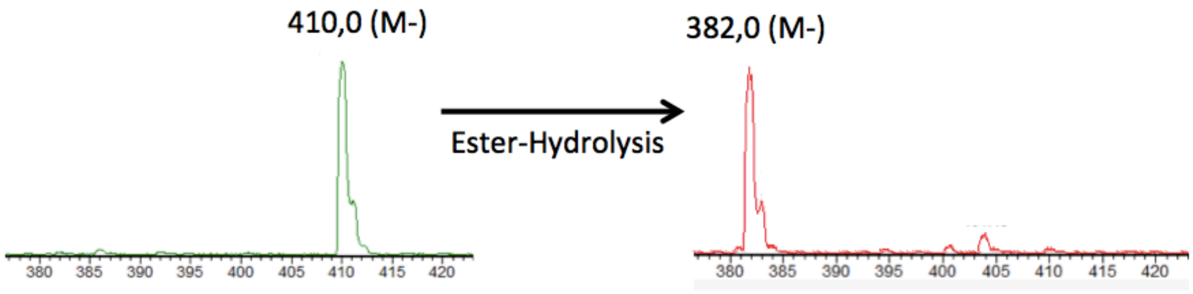

MS spectrum, operated in electrospray negative mode, of the prodrug AHU-377 (sacubitril, MW: 411) and the reaction product LBQ657 (sacubitrilat, MW: 383) following standard ester hydrolysis.

Supplementary Figure 6: Linearity of Angiotensin Metabolism Assay and Intra-Assay Variability

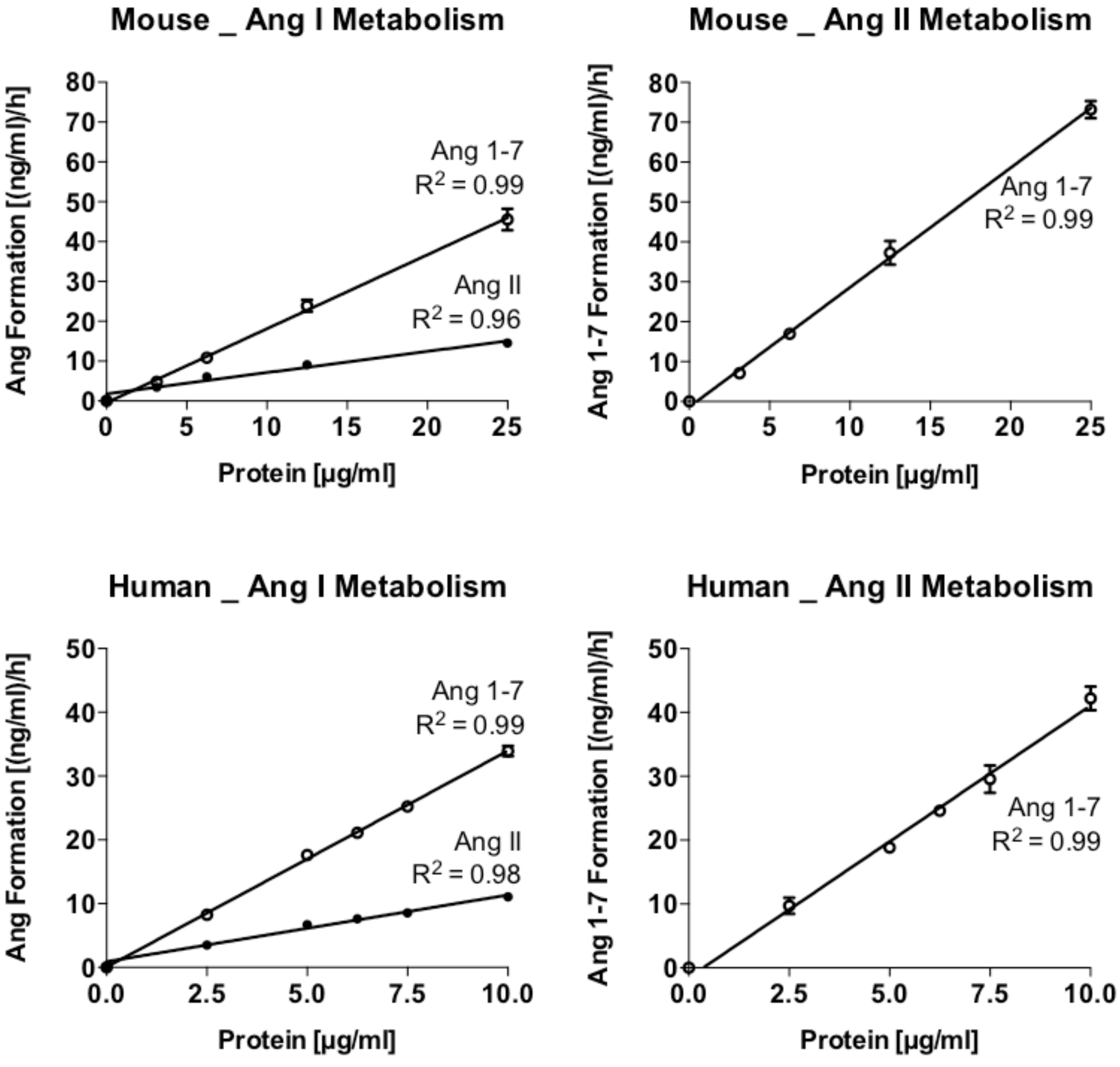

Increasing concentrations of homogenized murine or human kidney lysates (*n*=3) were spiked with Ang I and Ang II and the *ex vivo* Ang-(1-7) and AngII formation were monitored following incubation at 37 °C. Intra-assay variability at Ang I spike: Ang II = 4.31 %, Ang-(1-7) = 3.27 % Intra-assay variability at Ang II spike: Ang-(1-7) = 2.10 %. R^2 = Coefficient of correlation.
